# Supplementary figures and images for: Suppressing Pro-Apoptotic Proteins by siRNA in Corneal Endothelial Cells Protects against Cell Death
Source: Biomedicines. 2024 Jun 27;12(7):1439. doi: 10.3390/biomedicines12071439 (PMC11274739; doi:10.3390/biomedicines12071439)

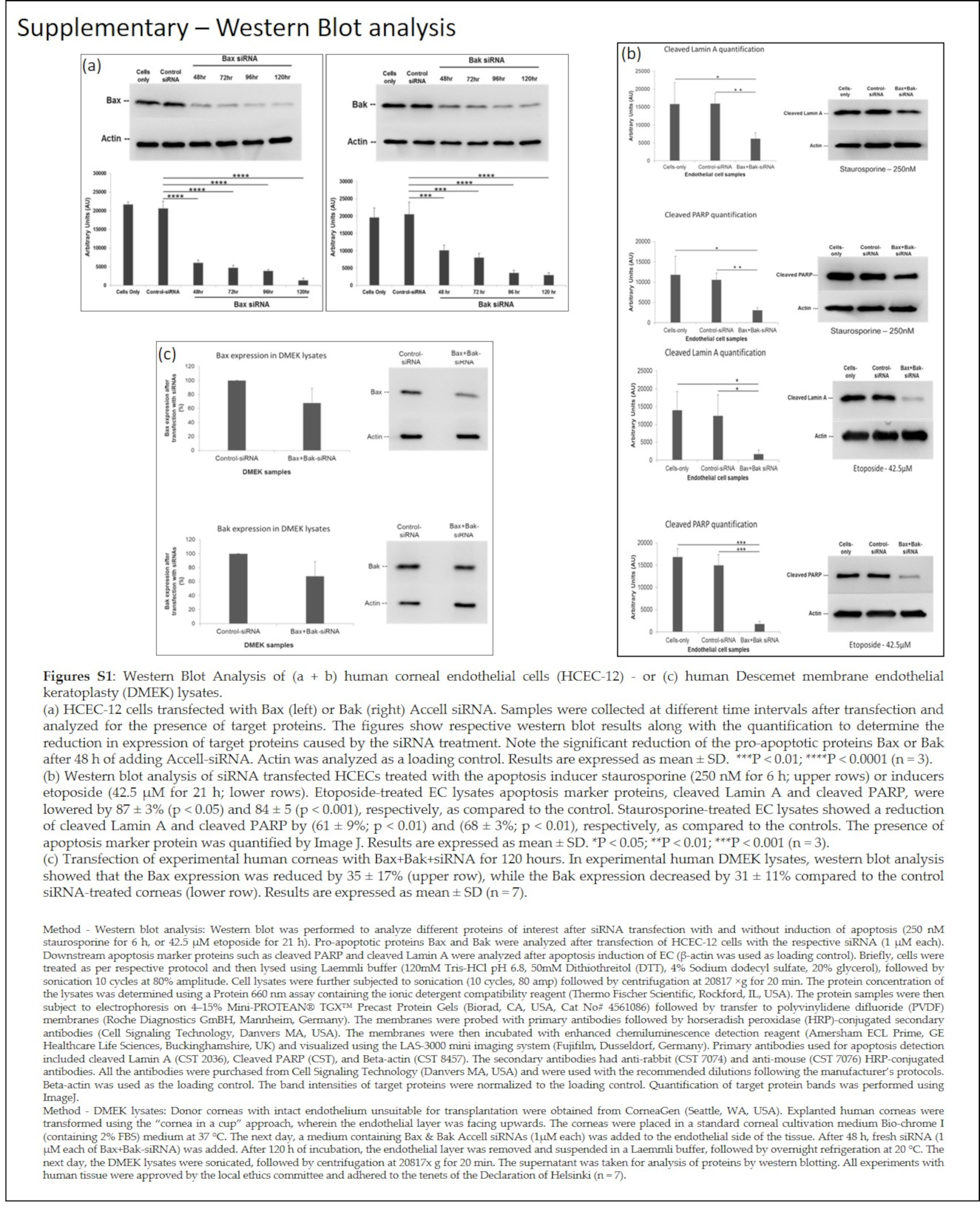

Supplement: Supplementary file 1 [file biomedicines-12-01439-s001.zip › biomedicines-3058990-supplementary.jpg]
